# „Jetzt Sprichst Du!“: Belastungen und psychosoziale Folgen der Coronapandemie für österreichische Kinder und Jugendliche

**DOI:** 10.1007/s00608-021-00909-2

**Published:** 2021-07-29

**Authors:** Manuel Schabus, Esther-Sevil Eigl

**Affiliations:** Laboratory for Sleep, Cognition, and Consciousness Research, Department of Psychology & Centre for Cognitive Neuroscience Salzburg (CCNS), Paris-Lodron University of Salzburg, Hellbrunnerstr. 34, 5020 Salzburg, Österreich

**Keywords:** Psychosoziale Beeinträchtigung, Kinder, Corona, Kollateralschäden, Psychische Gesundheit, Psychosocial burden, Children, Corona, Collateral damage, Mental health

## Abstract

Die Umfrage „Jetzt Sprichst Du!“ veranschaulicht eindrücklich die psychosozialen Belastungen und die Beeinträchtigung von Kindern und Jugendlichen in Österreich während der aktuellen Coronapandemie. Im Rahmen einer Online-Umfrage wurden 5483 Kinder und Jugendliche im Alter von 6 bis 18 Jahren bezüglich ihrer Gefühle, Ängste, Sorgen und Einschätzungen im Zusammenhang mit der Coronapandemie befragt. Es zeigt sich, dass die Kinder und Jugendlichen durch die Situation geängstigt sind und Mädchen hierbei über alle Altersgruppen hinweg stärker belastet sind. In diesem Zusammenhang wird auch deutlich, dass das Risiko einer COVID-19-assoziierten Hospitalisierung ähnlich wie bei Erwachsenen von den Kindern wie Jugendlichen massiv überschätzt wird. Auch macht sich in allen Altersgruppen eine hohe Perspektivenlosigkeit aufgrund der anhaltend herausfordernden Situation bemerkbar. Ferner häufen sich Gefühle von Wut, Ärger, Einsamkeit und Traurigkeit und es zeigt sich eine alarmierende Verschlechterung der Schlafqualität und eine Zunahme der Schlafproblematiken. Die Daten der Umfrage „Jetzt Sprichst Du!“ betonen die Notwendigkeit eines unabdingbaren und raschen Handelns, um sowohl die psychosozialen, entwicklungspsychologischen als auch gesundheitlichen Kollateralschäden in dieser jungen Altersgruppe einzudämmen, soweit dies heute noch möglich ist.

## Abstract (en)

The survey “Jetzt Sprichst Du!” (“Now you’re talking!”) impressively demonstrates the psychosocial burden and impairments of children and adolescents in Austria during the ongoing COVID-19 pandemic. As part of an online questionnaire, 5483 children and adolescents between 6 and 18 years of age were asked about their feelings, fears, worries, and assessments regarding the coronavirus pandemic. As can be seen, children and adolescents are worried about the situation, and girls are more burdened by this across all age groups. The study also clearly shows that the risk of a COVID-19-associated hospitalization is massively overestimated by children and adolescents, similar to adults. In addition, a great lack of perspective caused by the constantly challenging situation is evident across all age groups. Feelings of fury, anger, loneliness, and sadness are reported much more frequently than previously. Last but not least, our study shows an alarming reduction of sleep quality and a drastic increase in self-reported sleep problem already in this young population. The data from the “Jetzt Sprichst Du!” survey emphasize the need for inevitable and rapid action in order to limit the psychosocial and health-related collateral damage and to reduce the negative impact on young people’s psychological development, as far as this is still possible today.

## Hintergrund

Die Umfrage „Jetzt Sprichst Du!“ wurde am 21. Februar 2021 an der Universität Salzburg ins Leben gerufen, nachdem die psychosozialen Kollateralschäden der Coronapandemie und ihrer Maßnahmen zunehmend sichtbar wurden [1, 2]. Zudem ist es evident, dass gerade Kinder und Jugendliche von der Pandemie massiv beeinträchtigt [3] und in ihrer Entwicklung eingeschränkt werden [4] und ihre Stimmen bis dato wenig bis gar nicht gehört wurden [5].

Der Studie vorausgegangen war eine Umfrage zu Wissen und Einstellungen der Allgemeinbevölkerung hinsichtlich der Coronapandemie ab dem 18. Lebensjahr^1^.

## Methode

Konkret haben 5483 Kinder und Jugendliche zwischen 6 und 18 Jahren im Zeitraum vom 21.02.2021 bis 19.04.2021 an der österreichweiten Umfrage „Jetzt Sprichst Du!“^2^ der Universität Salzburg teilgenommen. Bekannt gemacht wurde die Umfrage über die österreichische Presseagentur (APA), den ORF und diverse Organisationen, die mit Kindern und Jugendlichen arbeiten.

Zur Datenbereinigung wurde vor der Auswertung der Daten neben dem Alter auch ein Plausibilitätscheck auf Datenintegrität durchgeführt. Im Speziellen wurde bei der Frage „Was denkst Du: Von 1000 Schülerinnen und Schülern, die so sind wie Du, wie viele davon werden in den nächsten 12 Monaten schwer an Corona erkranken und im Krankenhaus landen?“ und der damit assoziierten Frage „Wie groß schätzt Du die Gefahr für Dich persönlich ein, wegen Corona ins Krankenhaus zu müssen?“ geprüft, ob von Personen, die (i) angaben, dass maximal ein Fall (auf 1000) ins Krankenhaus muss, folgerichtig auch die subjektiv eingeschätzte Gefahr, wegen Corona ins Krankenhaus zu müssen, als „minimal“ oder „gar nicht“ eingestuft wurde. Zudem wurden Personen, die (ii) angaben, dass mindestens 412 von 1000 (95tes Perzentil) Kindern hospitalisiert werden müssten, nur dann in die finale Auswertung eingeschlossen, wenn sie zugleich ihr subjektives Risiko einer coronabedingten Hospitalisierung in diesem Sinn auch als „sehr groß“ oder „groß“ einschätzten. Die endgültige Stichprobengröße nach dieser Datenselektion ergab 5008 Kinder und Jugendliche zwischen 6 und 18 Lebensjahren, die die Umfrage bis zur letzten Frage des allgemeinen Teils beantworteten. Ein zweiter Teil der Umfrage konnte optional und zusätzlich ausgefüllt werden und umfasste Fragen zu Schlafgewohnheiten, Aktivitätsniveau und Smartphonegebrauch. Diesen Teil beendeten 2290 Kinder und Jugendliche. Er wird in einer gesonderten Veröffentlichung im Detail behandelt [6].

Bei der Auswertung der Fragen mit Rangreihen als Antwortoption wurden in der Analyse die Häufigkeiten der ersten 3 genannten Kategorien summiert bzw. angegeben. Wenn als Rang 1 bereits eine Kategorie gewählt wurde, die andeutete, dass keine weiteren Optionen zutreffend waren (z. B: „Ich habe eigentlich keine Angst“), wurde nur der erste Rang berichtet.

## Resultate

Von den 5008 Kindern und Jugendlichen waren 60,9 % weiblich, 37,9 % männlich und 1,2 % divers. Die Stichprobe wurde in 3 Altersgruppen eingeteilt: 6‑ bis 10-jährige Volksschüler (*n* = 949), 11- bis 14-jährige Mittelschüler (*n* = 1930) und 15- bis 18-jährige Jugendliche (*n* = 2129).

**Figure 1:**
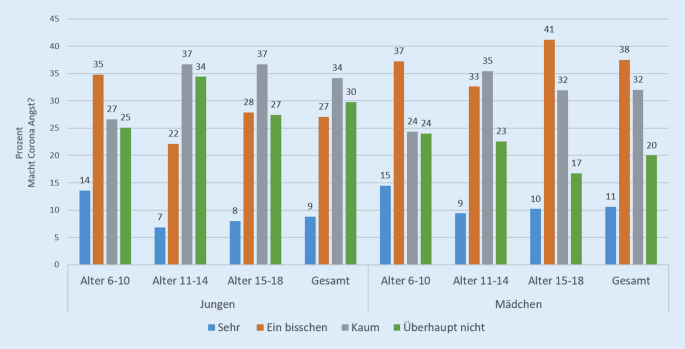

Auf die Frage, ob den Kindern und Jugendlichen „die aktuelle Situation mit Corona Angst“ mache, antworten 48,1 % der Mädchen und 35,9 % der Jungen, dass ihnen die Situation „sehr“ oder „ein bisschen“ Angst mache. Zwischen den Altersgruppen fällt auf, dass vor allem die Volksschüler die größten Ängste in Bezug auf Corona aufweisen und sich jedes zweite Kind durch die aktuelle Situation („sehr“ oder „ein bisschen“) verängstigt fühlt (Details siehe Abb. 1).

**Figure 2:**
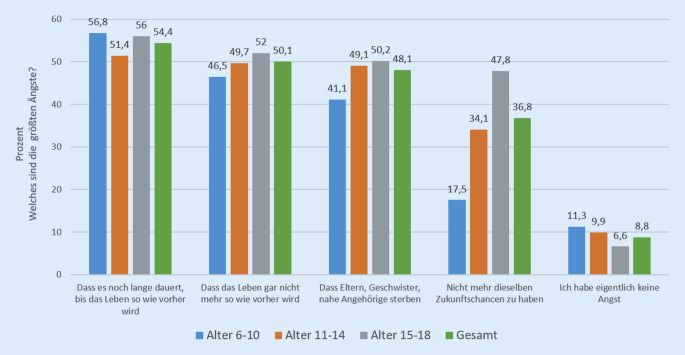

Fragt man nach der Art dieser Ängste, so werden in erster Linie folgende Befürchtungen genannt: „Dass es noch lange dauern wird, bis das Leben so wie vorher wird“ (54,4 %), „dass das Leben gar nicht mehr so wie vorher wird“ (50,1 %), „dass Eltern, Geschwister oder nahe Angehörige sterben“ könnten (48,1 %) sowie dass sie „nicht mehr dieselben Zukunftschancen bzw. Jobmöglichkeiten“ wie „vor Corona“ haben könnten (36,8 %); nur 8,8 % der Kinder und Jugendlichen gaben an, eigentlich keine dieser Ängste zu haben. Besonders bei den Jugendlichen ist die Angst, nicht mehr dieselben Zukunftschancen zu haben, deutlich ausgeprägt (47,8 %; vgl. Abb. 2).

**Figure 3:**
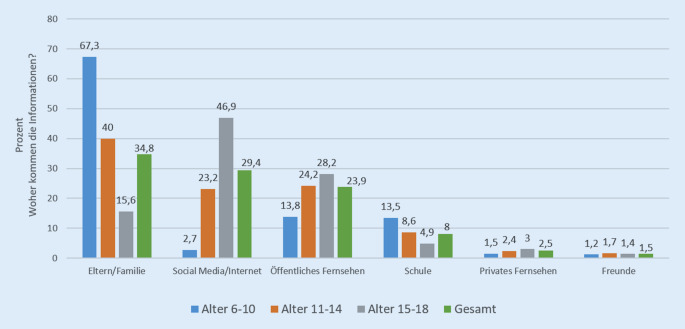

Als nächstes stellten wir uns die Frage, woher die Kinder und Jugendlichen hauptsächlich ihre Information über Corona beziehen. Auffällig ist, dass Volksschüler ihre Informationen bezüglich Corona primär über ihre Eltern und ihre Familie (67,3 %) beziehen, wohingegen Jugendliche primär Social Media oder das Internet als Informationsquelle nutzen (46,9 %); öffentliches Fernsehen und Schule spielen eine vergleichsweise untergeordnete Rolle (vgl. Abb. 3).

**Figure 4:**
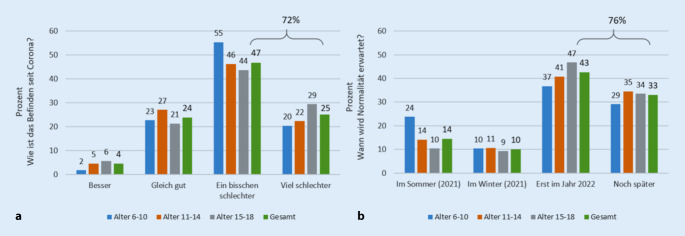

Fragt man die Kinder und Jugendlichen, wie es ihnen im Vergleich zu vor Corona geht, so geben 71,8 % an, dass es ihnen „viel“ (25 %) oder „ein bisschen“ (46,8 %) schlechter gehe als vor Corona (vgl. Abb. 4a). Fragt man, wann die Kinder und Jugendlichen denken, dass das „Leben wieder einigermaßen ‚normal‘ sein wird“, so sagen 75,6 %, dass sie eine Rückkehr zur Normalität erst im Jahr 2022 oder später erwarten (vgl. Abb. 4b). Dies ist Ausdruck einer gewissen Perspektivenlosigkeit der Kinder und Jugendlichen, die über alle Altersklassen beobachtet werden kann.

**Figure 5:**
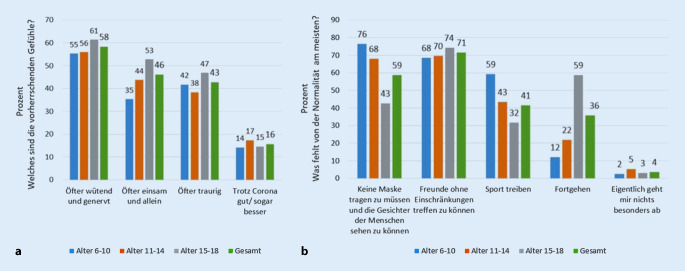

Ferner wurde nach den vorherrschenden Gefühlen seit Corona gefragt. Am häufigsten genannt wurde seit Corona „öfter wütend und genervt zu sein“ (58,2 %), gefolgt von „öfter einsam und allein zu sein“ (46 %) und „öfter traurig zu sein (42,7 %)“; 15,6 % fühlen sich „trotz Corona gut“ (13,6 %) oder „sogar besser“ (2 %; vgl. Abb. 5a). Fragt man nach den Dingen, die den Kindern und Jugendlichen von der Normalität am meisten fehlen, so wird am häufigsten angegeben „Freunde ohne Einschränkungen treffen zu können“ (71,4 %), gefolgt von „keine Masken tragen zu müssen und die Gesichter der Menschen sehen zu können“ (58,7 %) und „Sport treiben zu können“ (41,4 %). Die Jugendlichen geben „Fortgehen“ als zweithäufigste Antwortoption (58,7 %) an (vgl. Abb. 5b). Über alle Altersgruppen hinweg berichten die Kinder und Jugendlichen, dass ihnen der normale Schulalltag „extrem“ (29,8 %) bzw. „ziemlich“ (31,7 %) abgeht. Vor allem die Volksschüler leiden hier am meisten unter der aktuellen Situation mit 72,2 %, die angeben, dass ihnen der Alltag „sehr“ (42,1 %) oder „ziemlich“ (30,1 %) fehlt.

Laut Einschätzung der Kinder und Jugendlichen könne ihnen in der aktuellen Situation am meisten helfen „Freunde persönlich zu treffen“ (50,4 %), „Zeit mit der Familie“ (49,2 %) und „Zeit in der Natur“ (39,3 %) zu verbringen; 4,4 % geben an, dass ihnen eigentlich aktuell nichts hilft.

Ferner wurden die Kinder und Jugendlichen nach ihrer Einschätzung des Risikos, das vom Coronavirus (SARS-CoV-2) ausgeht, gefragt: „Was denkst Du: Von 1000 Schülerinnen und Schülern, die so sind wie Du, wie viele davon werden in den nächsten 12 Monaten schwer an Corona erkranken und im Krankenhaus landen?“. Interessanterweise wird das Risiko, wegen einer SARS-CoV-2-Infektion ins Krankenhaus zu müssen, von Kindern und Jugendlichen massiv überschätzt und mit 1,2–3,3 % beziffert, obwohl das Risiko in dieser jungen Altersgruppe unter 1 in 10.000 (< 0,01 %) Menschen mit Risikofaktoren bzw. unter 1 in 40.000 (< 0,003 %) bei Kindern bzw. Jugendlichen ohne Risikofaktoren liegt (vgl. Tab. 1).

**Table 1:** 

	Alter 6–10	Alter 11–14	Alter 15–18
5 % getrimmtes Mittel	12 (1,2 %)	33 (3,3 %)	31 (3,1 %)
QCovid-Risikobewertung (https://qcovid.org/) für eine COVID-19-assoziierte Hospitalisierung bei einer 19-jährigen, männlichen Person	1 in 43.478 (0,0023 %) ohne Risikofaktoren		
	1 in 10.753 (0,093 %) mit Diabetes Typ 1 und Asthma		

Der QCovid®-Algorithmus der Universität Oxford (UK) ist ein evidenzbasiertes Modell, das Faktoren wie Alter, Geschlecht, Ethnizität oder Vorerkrankungen einbezieht, um das Risiko einer COVID-19-assoziierten Hospitalisierung oder eines durch COVID-19 verursachten Todes abzuschätzen. Unter https://qcovid.org kann dieses Risiko für jedes Individuum errechnet werden. Als Veranschauungsbeispiel ist in dieser Tabelle das Risiko einer 19-jährigen, männlichen Person (180 cm, 80 kg) ohne Vorerkrankungen bzw. mit Diabetes Typ 1 und Asthma dargestellt und kann als obere Grenze verstanden werden

Auch die Angst, dass ein Elternteil oder naher Angehöriger stirbt – die eine der der primären Ängste der Kinder und Jugendlichen ist (Abb. 2) – scheint stark überhöht, nachdem das tatsächliche Risiko einer Hospitalisierung laut QCovid® selbst für die in den Medien oft strapazierten durchschnittlichen Großeltern (Modell gerechnet mit einer 75-jährigen Frau mit Diabetes Typ 2, Asthma, 165 cm, 70 kg) bei etwa 1 in 469 Fällen (0,21 %) und das des Versterbens bei etwa 1 in 1344 (0,07 %) Fällen liegt.

Außerdem haben wir versucht zu verstehen, ob es einen Unterschied macht, ob Kinder und Jugendliche in einem Umfeld mit auf Corona bezogen ängstlichen oder wenig ängstlichen Eltern leben. Hierfür wurde die Variable „Was denken Deine Eltern über Corona?“ zur Kategorisierung herangezogen und Kinder und Jugendliche, die der Meinung waren, die Eltern hielten Corona für „sehr gefährlich“ oder „gefährlich“, wurden der ängstlichen Gruppe zugeordnet. Kinder, die angaben, dass ihre Eltern Corona als „überhaupt nicht gefährlich“, „nicht sehr gefährlich“ oder „ähnlich gefährlich wie eine Grippe“ einschätzten, wurden als nicht ängstliche Gruppe definiert.

Über die Altersgruppen hinweg zeigt sich, dass die ängstliche Gruppe häufiger angab, ihre „Verwandten fast gar nicht mehr gesehen zu haben“ (44,6 % vs. 33,6 % bei der nicht ängstlichen Gruppe) oder etwas mehr Angst vor dem Virus zu haben (47,1 % vs. 40,1 %). Bei der Einschätzung der Gefährlichkeit des SARS-CoV-2-Erregers gab die ängstliche Gruppe zu 84 % an, Corona sei „sehr gefährlich“ oder „gefährlich“, wobei nur 13 % der nicht ängstlichen Gruppe dieser Einschätzung folgten. Auch schätzten 3‑mal mehr Kinder der ängstlichen Gruppe die Gefahr eines COVID-assoziierten Krankenhausaufenthalts als (sehr) groß ein (15,2 % vs. 4,7 %). Ein ähnliches Bild der Überschätzung des Risikos für eine Hospitalisierung ergibt sich im Rahmen einer Abfrage in absoluten Zahlen, wenngleich in einem anderen Ausmaß (4,28 % vs. 1,52 % Personen, „die genauso sind wie du“).

**Figure 6:**
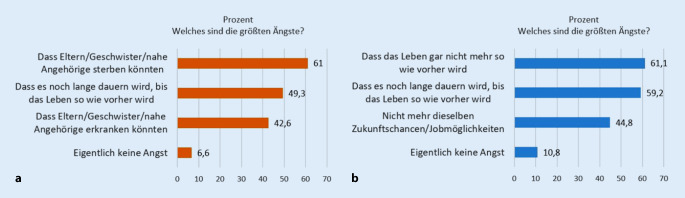

Auch die größten Ängste, die von den Kindern und Jugendlichen angegeben werden, unterscheiden sich in diesen beiden Gruppen maßgeblich. Wo die ängstliche Gruppe als primäre Befürchtung angibt, dass „Eltern, Geschwister oder nahe Angehörige sterben“ könnten (61 %) bzw. „dass Eltern, Geschwister oder nahe Angehörige erkranken“ könnten (42,6 %; vgl. Abb. 6a), beschäftigt die nicht ängstliche Gruppe vor allem, dass „das Leben gar nicht mehr so wie vorher“ werden könnte (61,1 %) bzw. dass sie „nicht mehr die dieselben Zukunftschancen bzw. Jobmöglichkeiten“ wie vor Corona haben könnten (44,8 %; vgl. Abb. 6b).

Vergleicht man diese beiden Gruppen nach ihren vorherrschenden Gefühlen seit Corona, zeigt sich das überraschende Bild, dass es die Kinder und Jugendlichen der nicht ängstlichen Gruppe sind, die häufiger angeben, sich seit Corona „öfter wütend und genervt“ zu fühlen (63,5 % vs. 52,6 %). Vermutlich ist es für diese Kinder schwer nachzuvollziehen, weshalb sie im Schulalltag eine Maske tragen müssen, Selbsttests durchführen sollen oder weshalb strenge Distanzregeln gelten und die somit einem erhöhten Druck im System ausgesetzt sind. Zudem zeigt sich, dass das Gefühl, sich „öfter einsam und allein zu fühlen“, vom Volksschulalter bis hin zur Gruppe der Jugendlichen merkbar ansteigt. Hier liegt die Vermutung nahe, dass dies damit zusammenhängt, dass es die Coronamaßnahmen den Jugendlichen nicht oder nur bedingt erlauben, ihre Freunde und Altersgenossen zu treffen, wohingegen Volksschulkinder ohnehin noch mehr Zeit in der Familie selbst verbringen (vgl. Tab. 2).

**Table 2:** 

**„Ängstlich“**	**Gesamt**	**Alter 6–10**	**Alter 11–14**	**Alter 15–18**
	*n* *=* *2456*	*n* *=* *383*	*n* *=* *922*	*n* *=* *1151*
Ich bin öfter traurig als vor Corona	39,8	37,6	36,6	43,0
Ich fühle mich öfter einsam und allein	44,9	34,1	42,6	50,5
Ich fühle mich öfter wütend und genervt	52,6	43,8	49,8	57,9
Ich fühle mich trotz Corona gut oder sogar besser^a^	19,4	19,6	21,0	18,1
**„Nicht ängstlich“**	**Gesamt**	**Alter 6–10**	**Alter 11–14**	**Alter 15–18**
	*n* *=* *2546*	*n* *=* *566*	*n* *=* *1004*	*n* *=* *976*
Ich bin öfter traurig als vor Corona	45,4	44,4	40,0	51,4
Ich fühle mich öfter einsam und allein	47,1	36	45,0	55,5
Ich fühle mich öfter wütend und genervt	63,5	63,1	61,7	65,6
Ich fühle mich trotz Corona gut oder sogar besser^a^	12,0	10,6	13,9	10,7

^a^Akkumulierung der Antwortoptionen „Ich fühle mich trotz Corona gut“ und „Ich fühle mich sogar besser als zuvor“

Im zweiten Teil des Fragebogens, der hier nur exemplarisch behandelt wird (*n* = 2290), fällt auf, dass die körperlichen Aktivitäten seit Corona bei 74,8 % der Kinder und Jugendlichen mit „sehr viel weniger“ oder „weniger“ angegeben werden. Zudem wird auch die Zeitspanne, die die Teilnehmer unter natürlichem Tageslicht verbringen, kürzer. Sie zeigt sich bei 44,2 % der Teilnehmer als reduziert. Hingegen verbringen die Kinder wie auch Jugendlichen wesentlich mehr Zeit mit Smartphone, Tablet oder PC (85,0 %). Unter diesen Umständen hat sich auch das Schlafverhalten seit Corona unter den Kindern und Jugendlichen stark verändert.

Mehr als jedes drittes Kind (37 %) gibt an, seit Corona eine schlechtere Schlafqualität aufzuweisen; 38,9 % der Kinder berichten sogar, nun Probleme mit dem Schlaf zu haben, was für dieses junge Alter (6.–18. Lebensjahr) äußerst unüblich ist. Von denjenigen, die über diese Schlafprobleme berichten, sind 42,5 % von Einschlafschwierigkeiten und 20,3 % von Durchschlafschwierigkeiten geplagt. Außerdem gehen 9 von 10 Kindern bzw. Jugendlichen (94,3 %) nun während der Woche später zu Bett. Altersspezifisch zeigen sich die meisten coronabedingten Schlafprobleme bei den Jugendlichen (45,3 %) und eine Häufung von Albträumen bei den Jüngsten (16,3 %).

## Resümee

Zusammenfassend zeichnet sich ein alarmierendes Bild der psychosozialen Gesundheit bei Kindern und Jugendlichen ab und verdeutlich den akuten Handlungsbedarf. Die besonders eindringlichen und noch nicht veröffentlichten (anonymen) Kommentare der Kinder und Jugendlichen der Umfrage „Jetzt Sprichst Du!“ zeichnen hier ein besonders deutliches Bild^3^.

Als sinnbildliche Handlungsempfehlung wollen wir Bewegung und Begegnung ausgeben. Leider sind es ausgerechnet die Faktoren, die den Kindern und Jugendlichen gut durch die Pandemie helfen würden, die aufgrund der Coronamaßnahmen unmöglich gemacht werden oder nur sehr bedingt möglich sind.

Aus unserer Sicht muss es daher erste Priorität sein, sich um eine rasche Normalisierung inklusive des Freizeit- und Sportangebots für Kinder und Jugendliche zu bemühen und im Sinn der Prävention schon heute tätig zu werden. Die Auswirkungen des oft zitierten „verlorenen Jahres“ für Kinder und Jugendliche sind sowohl aus psychologischer als auch psychotherapeutischer Sicht in ihrem Ausmaß heute nur schwer abschätzbar. Es ist aber wohl unbestritten, dass sie massiv sein werden und sich sowohl in psychosomatischen Erkrankungen [7] als auch in Verhaltensauffälligkeiten wie Schulverweigerung widerspiegeln werden. In diesem Sinn wäre es auch angezeigt, schon heute an den Ausbau von schulpsychologischen wie auch pädagogischen und psychotherapeutischen Angeboten für Kinder und Jugendliche zu denken.

Persönlich befürchten wir, dass im psychosozialen Sektor das Schlimmste noch bevorstehen könnte. Dann nämlich, wenn sich die wirtschaftlichen Folgen der Pandemie inklusive erhöhter Arbeitslosigkeit zeigen und somit auch der Stress in den Familien steigt, ist auch mit vermehrter Gewalt in Familien [8] und in der Folge mit psychischen Reaktionen der Kinder und Jugendlichen zu rechnen [9].

Aus unserer Sicht ist daher klar: Wir müssen heute handeln, um nicht morgen vor den Scherben unserer Versäumnisse zu stehen und eine verlorene Generation verantworten zu müssen.

## Footnotes

1 Vgl. Medienberichte auf www.sleepscience.at.

2 Der Fragebogen ist auf http://bit.ly/JetztSprichstDu_supplements im Detail abzurufen.

3 Siehe http://bit.ly/JetztSprichstDu_supplements.
